# Do we cause false positives? An experimental series on droplet or airborne SARS-CoV-2 contamination of sampling tubes during swab collection in a test center

**DOI:** 10.1186/s13756-021-00920-z

**Published:** 2021-03-09

**Authors:** Thomas Scheier, Cyril Shah, Michael Huber, Hugo Sax, Barbara Hasse, Huldrych F. Günthard, Alexandra Trkola, Peter W. Schreiber

**Affiliations:** 1 Division of Infectious Diseases and Hospital Epidemiology, University Hospital Zurich and University of Zurich, Zurich, Switzerland; 2 Institute of Medical Virology, University of Zurich, Zurich, Switzerland

**Keywords:** SARS-CoV-2, COVID-19, False-positive test results, Contamination

## Abstract

The rapid spread of the coronavirus disease 2019 pandemic urged immense testing capacities as one cornerstone of infection control. Many institutions opened outpatient SARS-CoV-2 test centers to allow large number of tests in comparatively short time frames. With increasing positive test rates, concerns for a possible airborne or droplet contamination of specimens leading to false-positive results were raised. In our experimental series performed in a dedicated SARS-CoV-2 test center, 40 open collection tubes placed for defined time periods in proximity to individuals were found to be SARS-CoV-2 negative. These findings argue against false-positive SARS-CoV-2 results due to droplet or airborne contamination.

## Introduction

In December 2019, a novel Coronavirus, later named severe acute respiratory syndrome coronavirus 2 (SARS-CoV-2), was detected as causative pathogen in a cluster of pneumonia of unknown cause  in Wuhan, China [1]. Since then, multiple assays for the detection of SARS-CoV-2 were established, enabling diagnosis of coronavirus disease 2019 (COVID-19). Besides nucleic acid amplification tests, antigen-based tests and serological assays are now available [2, 3]. An increasing demand for test capacities due to the pandemic spread prompted implementation of outpatient SARS-CoV-2 test centers. Considering the high sensitivity of SARS-CoV-2 polymerase chain reaction (PCR) used in highly frequented test center settings, concerns of false-positive PCR results due to contamination during pre-analytic processes occurred. The objective of the present study was to investigate, whether sampling tubes can be inadvertently contaminated with SARS-CoV-2 by droplets or aerosols generated by SARS-CoV-2 infected individuals during nasopharyngeal swab (NPS) sampling.

## Material and methods

### Setting

This study was performed at the SARS-CoV-2 test center of the University Hospital Zurich (USZ), Zurich, Switzerland. The center serves as referral center for all outpatient clinics of the USZ and is open for public. All individuals attending the SARS-CoV-2 test center are obliged to wear nose-mouth masks and to perform an alcohol-based hand disinfection at entry. Healthcare worker (HCW) register the personal data and assess symptoms compatible with COVID-19 using a standardized questionnaire before testing. A physical examination is not performed. During the swab procedure the nose-mouth mask is temporarily removed. After gathering the nasopharyngeal swab, individuals leave the test center directly and the results are provided by a phone call or short message service. In normal operation, the room is throughout ventilated by a tilted window. Between consecutive tests, the exterior doors are opened and all surfaces that were in contact with the tested individual are disinfected. Health care workers performing the swab procedure are wearing gloves, gowns, surgical masks (type 2R) and googles. Gloves are either changed or disinfected (for a maximum of five times) after every patient. Gowns are changed at the end of the shift, or if the assigned rooms for swab collection are left.

### Experimental set-up

**Fig. 1:**
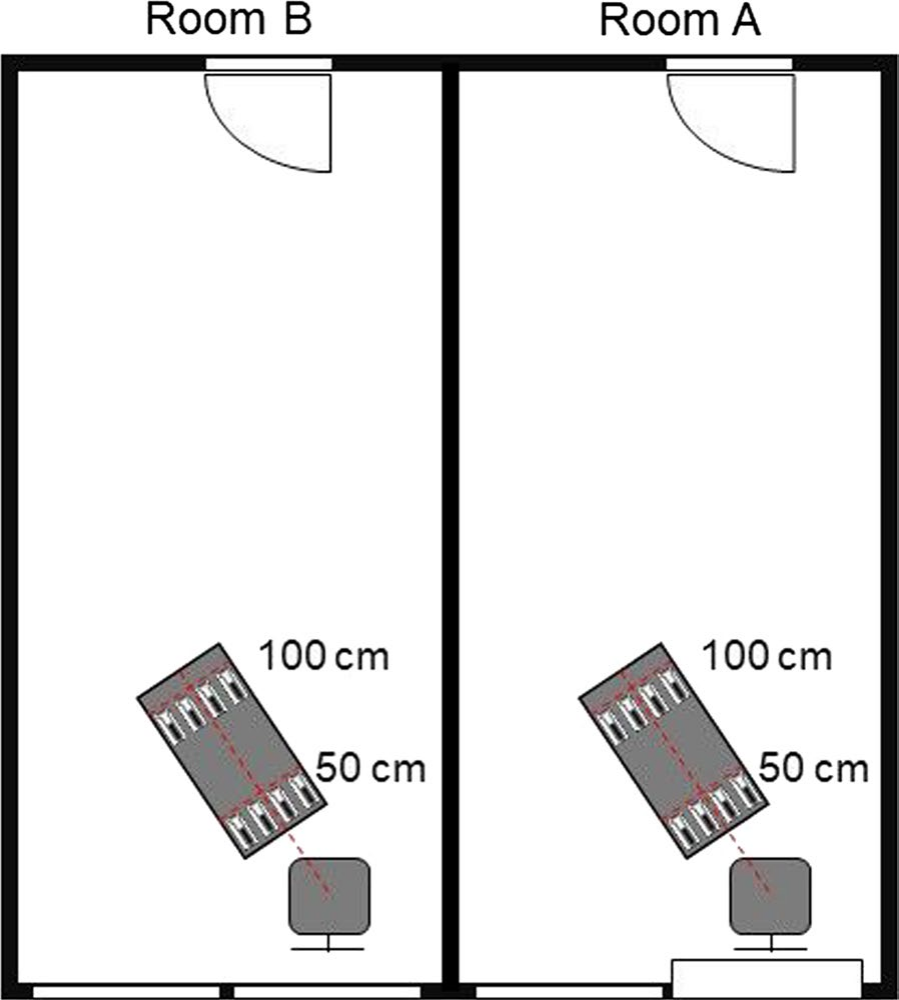
Experimental set-up. Room A: size of 18.96m^2^, window (at the back of the chair, on which individuals were seated; window area of around 1.7 m^2^) was permanently tilted throughout experimental series. Room B: size of 19.88m^2^, window was closed during an experimental series, after each experimental series the window (at the back of the chair, on which individuals were seated; window area of around 1.7 m^2^) was tilted for approximately 10 min prior to the next experimental series

The experimental series was performed in two adjacent rooms designated for individuals with COVID-19 compatible symptoms or a history of relevant SARS-CoV-2 exposure (Fig. 1). Both rooms featured a similar room size (room A 18.96 m^2^ and room B 19.88 m^2^) and window area (room A and B around 1.7 m^2^). Both rooms have horizontal pivot windows. Individuals that presented for SARS-CoV-2 testing were seated in front of the window for NPS collection. In room A, the window was permanently tilted for continuous air exchange. In room B, the window was closed during the experimental runs, each lasting 60 min. Then, the window was tilted for about 10 min before launching the next experimental run. Both rooms featured no ventilation systems. In each room, three open tubes (15 ml, opening diameter 17 mm) filled with 3 ml viral transport medium (VTM) were placed at a distance of 50cm and 100cm to the patient. At each distance, one tube was sealed after 5, 10 and 60 min, respectively. This procedure was repeated for a total of three times. In addition, at both distances one separate open tube filled with 3 ml VTM was placed at the beginning of the first experimental series and was closed at the end of the third experimental series. To avoid contact transmission, HCW performed a hygienic hand disinfection routinely prior to handling of virus sample tubes. The overall number of persons that received SARS-CoV-2 testing in these rooms during the experimental series was registered. Results of the SARS-CoV-2 tests and the corresponding cycle thresholds (cT) values were retrieved from the electronic patient information system.

### SARS-CoV-2 polymerase chain reaction

All samples were analyzed at the Institute of Medical Virology of the University of Zurich. SARS-CoV-2 PCR analyses were performed with Roche Cobas SARS-CoV-2 IVD test (Roche Diagnostics, Mannheim, Germany) and an in-house high input PCR. Briefly, 100 µl of sample were lysed with 50 µl AVL buffer (Qiagen, Venlo, the Netherlands), precipitated with 150 µl EtOH and centrifuged at 21,000*g* for 1 min. The supernatant was removed, and the pellet resuspended with 35 µl of water. RT-qPCR was performed targeting the E- and the N-gene according to Corman et al*.* [4] with 16 µl eluate each using the TaqMan Fast Virus 1-Step Master Mix (Thermo Fisher, Waltham, MA, USA).

## Results

At the day of the experiments, totally 254 persons presented at the SARS-CoV-2 test center. Among these, 47 individuals tested positive, corresponding to a positivity rate of 18.5%. During the experimental series, 39 persons were tested for SARS-CoV-2 in room A and 41 persons in room B, respectively. In both rooms, the number of persons that received NPS sampling was similar for all predefined sampling periods (detailed information is provided in Table 1). Among the 80 individuals that were tested in either room A or room B, 19 (23.8%) tested positive. The median cT value of SARS-CoV-2 positive individuals was 22.38 (interquartile range (IQR) 19.24–25.73) for open reading frame 1 (ORF) and 22.68 (IQR 18.93–25.87) for envelope protein gene (E-gene), respectively. All 40 environmental samples tested negative for SARS-CoV-2 in both PCR methods applied.

**Table 1:** Number of persons that received SARS-CoV-2 testing during experimental series

Experimental series	Duration (min)	Number of tested persons	
		Room A	Room B
1	5	2 (+ 2)	2 (+ 2)
1	10	3 (+ 1)	2 (+ 0)
1	60	12 (+ 9)	13 (+ 11)
2	5	2 (+ 2)	2 (+ 2)
2	10	2 (+ 0)	2 (+ 0)
2	60	8 (+ 6)	9 (+ 7)
3	5	2 (+ 2)	2 (+ 2)
3	10	3 (+ 1)	3 (+ 1)
3	60	19 (+ 16)	19 (+ 16)
Total		39	41

In the column “Number of tested persons”, the number indicates the cumulative number of individuals that received SARS-CoV-2 testing during the experimental series; the number in brackets corresponds to the number of additionally tested individuals compared to the previous time period

In experimental series 1, 4/25 (16.0%) individuals, in experimental series 2, 7/17 (41.2%) individuals and in experimental series 3, 8/38 (21.1%) individuals tested SARS-CoV-2 positive, respectively

## Discussion

The present study addressed the question whether false-positive results for SARS-CoV-2 could result from droplet or airborne contamination originating from individuals that have been sampled previously in the same room. Our experimental series encompassed various exposure times, two different settings regarding ventilation and two PCR assays, including a commonly used assay for SARS-CoV-2 diagnostics and a highly sensitive high input PCR, did not detect any contamination of open sampling tubes with SARS-CoV-2.

Prior studies reported contamination of several surfaces with SARS-CoV-2 next to infectious individuals [5]. Surroundings of infected individuals can either get contaminated by SARS-CoV-2 containing droplets, which is considered as by far the most relevant transmission mode, or exhaled aerosols or via contact transmission, e.g. if devices are touched after hands have been contaminated during coughing. The estimated median half-life of viable SARS-CoV-2 on different materials in an experimental setting was about 6.8 h and 5.6 h on plastics and stainless steel, respectively [6]. The high proportion of COVID-19 affected individuals in dedicated test centers promotes environmental contamination with SARS-CoV-2, and the level of contamination presumably increases with proximity to infected persons. As detailed above, the main transmission mode of SARS-CoV-2 occurs via droplets expelled during talking, coughing or sneezing [3]. For smaller droplets or aerosols, which can be generated during coughing by some individuals, ventilation becomes relevant for clearance [3, 7]. Our experimental set-up included a room without any enhanced air exchange and a room with continuous air exchange via tilted windows. Independent of the set-up all samples tested negative, even in a high input PCR with increased sensitivity.

Our study has some limitations. We can only provide information on the number of individuals that tested SARS-CoV-2 positive during the experimental series in both rooms. We did not register which patients were assigned to either room A or B. Nevertheless, as all samples tested negative, the assignment to the individual room does not seem crucial for the interpretation of the results. In addition, we did not swab surfaces to test for environmental contamination. However, considering the strict application of personal protective equipment, hand hygiene and surface disinfection, contamination around infected individuals is likely very limited. Strict application of hand hygiene and glove change of HCWs were likely crucial to prevent contamination of virus sample tubes.

In conclusion, we could not detect SARS-CoV-2 contamination of open collection tubes during NPS collection, even if these specimens remained unsealed for more than 3 h. Stringent use of personal protective equipment, including wearing of a surgical mask by the individual to be tested as a component of source control, hand hygiene and regular surface disinfection prevented successfully contamination. Furthermore, adequate specimen handling includes immediate closure after insertion of the NPS, thus minimizing the risk of contamination.

## Data Availability

The datasets used and/or analysed during the current study are available from the corresponding author on reasonable request.

## Abbreviations

COVID-19: Coronavirus disease 2019
cT: Cycle threshold
E-Gene: Envelope protein gene
HCW: Healthcare worker
NPS: Nasopharyngeal swab
ORF: Open reading frame 1
PCR: Polymerase chain reaction
SARS-CoV-2: Severe acute respiratory syndrome coronavirus 2
USZ: University Hospital Zurich
VTM: Viral transport medium

## References

[1] World Health Organization. Rolling updates on coronavirus disease (COVID-19), Updated 31 July 2020. https://www.who.int/emergencies/diseases/novel-coronavirus-2019/events-as-they-happen.

[2] World Health Organization. Diagnostic testing for SARS-CoV-2. 2020.

[3] Wiersinga WJ, Rhodes A, Cheng AC, Peacock SJ, Prescott HC (2020). Pathophysiology, transmission, diagnosis, and treatment of coronavirus disease 2019 (COVID-19): a review. JAMA.

[4] Corman VM, Landt O, Kaiser M, Molenkamp R, Meijer A, Chu DK (2020). Detection of 2019 novel coronavirus (2019-nCoV) by real-time RT-PCR. Euro Surveill.

[5] Ong SWX, Tan YK, Chia PY, Lee TH, Ng OT, Wong MSY (2020). Air, surface environmental, and personal protective equipment contamination by severe acute respiratory syndrome coronavirus 2 (SARS-CoV-2) from a symptomatic patient. JAMA.

[6] van Doremalen N, Bushmaker T, Morris DH, Holbrook MG, Gamble A, Williamson BN (2020). Aerosol and surface stability of SARS-CoV-2 as compared with SARS-CoV-1. N Engl J Med.

[7] Somsen GA, van Rijn C, Kooij S, Bem RA, Bonn D (2020). Small droplet aerosols in poorly ventilated spaces and SARS-CoV-2 transmission. Lancet Respir Med.

